# Recurrence and survival rates of inflammatory bowel disease-associated colorectal cancer following postoperative chemotherapy: a comparative study

**DOI:** 10.1093/gastro/gow016

**Published:** 2016-06-08

**Authors:** Mohannad Dugum, Jingmei Lin, Rocio Lopez, Bassam Estfan, Elena Manilich, Luca Stocchi, Bo Shen, Xiuli Liu

**Affiliations:** 1 Division of Gastroenterology, Hepatology and Nutrition, Department of Medicine, University of Pittsburgh, Pittsburgh, PA, USA; 2 Department of Pathology and Laboratory Medicine, Indiana University School of Medicine, Indianapolis, IN, USA; 3 Quantitative Health Sciences, Cleveland Clinic, Cleveland, OH, USA; 4 Department of Hematology and Oncology, Taussig Cancer Institute, Cleveland Clinic, Cleveland, OH, USA; 5 Department of Colorectal Surgery, Digestive Disease Institute, Cleveland Clinic, Cleveland, OH, USA; 6 Department of Gastroenterology and Hepatology, Digestive Disease Institute, Cleveland Clinic, Cleveland, OH, USA; 7 Department of Anatomic Pathology, Pathology & Laboratory Medicine Institute, Cleveland Clinic, Cleveland, OH, USA

**Keywords:** inflammatory bowel disease, colorectal cancer, adjuvant chemotherapy, prognosis

## Abstract

**Background and Aim**: Inflammatory bowel disease (IBD) is associated with an increased risk of colorectal cancer (CRC). Studies have shown tumorigenetic and histomorphological differences between IBD-associated CRC and non-IBD CRC, suggesting differences in tumor behavior and response to treatment. We aimed to compare tumor recurrence and survival rates following postoperative chemotherapy in CRC patients with and without IBD.

**Methods:** Search of the Cleveland Clinic’s CRC database revealed 65 patients who had IBD-associated CRC and received postoperative adjuvant chemotherapy between 1994 and 2010. Twenty-one patients were excluded due to incomplete clinical data. Propensity score-matching based on age, surgery intent, CRC site, tumor grade, American Joint Committee on Cancer (AJCC) stage and T stage was used to match IBD and non-IBD patients (1:4). Competing risk and Cox regression models were used to analyze differences in disease-free survival and overall survival, respectively.

**Results:** Forty-four patients with IBD-associated CRC were matched to 176 patients with non-IBD CRC. Among IBD patients, 29 (66%) had ulcerative colitis, 14 (32%) had Crohn’s disease, and one (2%) had indeterminate colitis. Mean IBD diagnosis age was 28.1 ± 14.5 years, and mean IBD duration at time of CRC treatment was 21.5 ± 12.6 years. Ten (23%) IBD patients had tumor recurrence compared with 34 (19%) non-IBD patients (*P* = .074). There was no significant difference in disease-free survival (hazard ratio [HR] = 0.60; 95% CI: 0.35–1.05; *P* = 0.074) or overall survival (HR = 0.87; 95% CI: 0.54–1.4; *P* = 0.58) between IBD and non-IBD patients.

**Conclusion:** Patients with IBD-associated CRC have comparable rates of tumor recurrence and survival following postoperative chemotherapy as CRC patients without IBD. Prospective studies are needed to confirm these findings and guide therapeutic decisions.

## INTRODUCTION

Colorectal cancer (CRC) is currently the third most common cancer in both males and females, and the second leading cause of cancer-related deaths in the United States [1]. Worldwide, CRC is responsible for about 600, 000 deaths every year [2]. While most cases of CRC are sporadic, 1–2% of CRC patients have underlying inflammatory bowel disease (IBD), either ulcerative colitis (UC) or Crohn’s disease (CD). IBD patients have an increased risk of developing CRC compared with the general population, and IBD-associated CRC tends to be difficult to treat and carries a higher mortality rate compared with sporadic CRC [3,4]. The overall prevalence of CRC in UC patients with pancolitis is in the range of 4–5%, with a similar percentage observed in CD patients who have severe, long-standing colitis in the absence of bowel resection [5].

The association between CRC and IBD is well established in patients with colitis due to either UC or CD [6]. Specific molecular signaling pathways have been linked to the development of sporadic CRC, while additional pathways—including unique ones—have been implicated in the carcinogenesis of IBD-associated CRC [7]. IBD patients tend to have excessive inflammatory cell infiltration and increased expression of a number of inflammatory genes [8]. This mucosal inflammation promotes cellular proliferation and ultimately the development of CRC. Further evidence for the inflammation-to-cancer cascade came from studies demonstrating a protective role for anti-inflammatory medications such as non-steroidal anti-inflammatory drugs and 5-aminosalicylic acid, which were shown to reduce the risk of colitis-related CRC [9].

Given the tumorigenetic and histomorphological differences between IBD-associated and sporadic CRC, differences in tumor behavior could be present and potentially affect the response to treatment. The aim of this study was to assess differences in tumor recurrence and survival rates following postoperative chemotherapy in CRC patients with and without IBD in order to determine the potential need for tailored management of patients with IBD-associated CRC.

## PATIENTS AND METHODS

### Study population

After obtaining institutional review board approval, the Cleveland Clinic’s prospectively maintained CRC database was accessed. Adult patients were identified who had been diagnosed with CRC, underwent surgical treatment for CRC and subsequently received postoperative chemotherapy between 1994 and 2010. Baseline patient characteristics including the presence or absence of underlying IBD were recorded. Patients with incomplete clinical data were excluded. All patients were staged according to the 7th edition of the American Joint Committee on Cancer (AJCC) staging system [10].

### Patient matching

A propensity score was used to match each patient with IBD-associated CRC to 4 non-IBD patients with CRC. A logistic regression model was used to create the propensity score, which included the following variables: age at time of surgical treatment, intent of surgery (curative *vs* palliative), site of CRC (colon, rectum), grade of tumor differentiation, AJCC stage and T stage. The probability of having IBD was used as the propensity score, and a greedy matching algorithm was used to find the best matches for each IBD patient. Median difference in propensity score between matches was 0.001 (25th, 75th percentiles: 0.001, 0.02).

### Statistical analysis

Mean ± standard deviation or median (25th, 75th percentiles) were used for continuous variables and N (%) for categorical factors. A univariable analysis was performed to assess differences between CRC patients with and without IBD. Analysis of variance or non-parametric Kruskal-Wallis tests were used for continuous or ordinal variables, and Pearson chi-square tests were used for categorical factors. A time-event-analysis was used to assess overall survival and disease-free survival following surgical treatment of CRC. Follow-up time for overall survival was defined as months from surgery to death or last follow-up if event-free at end of the follow-up period. Follow-up time for disease-free survival was defined as months from surgery to disease recurrence, disease-unrelated death or last follow-up if event-free at end of follow-up period.

Kaplan-Meier plots were constructed, and regression models were used to compare the groups while adjusting for the propensity score. Competing risk and multivariable Cox regression models were used to analyze the differences in disease-free survival and overall survival, respectively, between CRC patients with and without IBD. For the competing risks analysis, patients were censored if they died from other reasons or if they remained alive at the last follow-up. A *P* value < 0.05 was considered statistically significant. All analyses were performed using SAS version 9.2 (SAS Institute, Cary, NC) and R version 3.0.2 (R Foundation for Statistical Computing, Vienna).

## RESULTS

A total of 65 patients with IBD-associated CRC who received postoperative chemotherapy were identified. After excluding 21 patients due to lack of complete clinical data; 44 patients with IBD-associated CRC who received postoperative chemotherapy were matched to 176 non-IBD patients with CRC who received postoperative chemotherapy. The form of chemotherapy prior to 2004 was fluorouracil, while most patients treated after 2004 received both fluorouracil and oxaliplatin.

Among 220 patients with CRC, 163 (75%) had colon cancer, and 57 (25%) had rectal cancer. Of the IBD-associated CRC patients, 10 (23%) had tumor recurrence (3 local, 6 distant, 1 local and distant) compared with 34 (19%) of non-IBD patients with CRC (2 local, 25 distant, 7 local and distant) (*P* = 0.074). **Table 1** summarizes the characteristics of all CRC patients, comparing patients with IBD-associated CRC with matched non-IBD CRC patients.

**Table 1.** Patient characteristics

	Non-IBD CRC (N = 176)	IBD-associated CRC (N = 44)	*P* value
Age at time of surgery (years)	51.4 ± 12.5	50.1 ± 13.0	0.55^a^
Male	131 (74)	32 (73)	0.82^c^
Intent of surgery			0.77^c^
Curative	151 (86)	37 (84)	
Palliative	25 (14)	7 (16)	
Site of CRC			0.88^c^
Colon	130 (74)	33 (75)	
Rectal	46 (26)	11 (25)	
AJCC stage			0.99^b^
I	4 (2)	1 (2)	
II	29 (17)	8 (18)	
III	118 (67)	28 (64)	
IV	25 (14)	7 (16)	
T stage			0.79^b^
T1	6 (3)	2 (5)	
T2	27 (15)	5 (11)	
T3	119 (68)	31 (70)	
T4	24 (14)	6 (14)	
Grade of differentiation			0.54^b^
Well	3 (2)	1 (2)	
Moderate	95 (54)	21 (48)	
Poor	78 (44)	22 (50)	
Follow-up (months)	64.8 ± 71.0	74.1 ± 58.9	0.42^a^
Propensity score^†^	0.85 ±0.08	0.83 ± 0.10	0.14^a^

Values presented as mean ± standard deviation or number  (column %).

*P* values:

a = ANOVA,

b = Kruskal-Wallis test,

c = Pearson chi-square test.

† A logistic regression model was used to create the propensity score: age at time of surgical treatment, intent of surgery, site of CRC, grade of tumor differentiation, AJCC stage and T stage.

Of the IBD patients, 29 (66%) had UC, 14 (32%) had CD, and 1 (2%) had indeterminate colitis. All IBD-associated CRC cases were confirmed by histologic review. Thirty-six patients had available data on IBD treatment, and 26 of them (72%) were receiving medical treatment for IBD at the time of CRC diagnosis. Six patients (14%) had synchronous primary sclerosing cholangitis (all with UC). Average age at time of IBD diagnosis was 28.1 ± 14.5 years, and duration of IBD at time of CRC treatment was 21.5 ± 12.6 years. **Table 2** summarizes the characteristics of patients with IBD-associated CRC.

**Table 2.** IBD-associated CRC patient characteristics

	Total (N = 44)
Age at IBD diagnosis (years)	28.1 ± 14.5
Type of IBD	
UC	29 (66)
CD	14 (32)
Indeterminate colitis	1 (2)
IBD activity at time of CRC diagnosis	
Active	19 (43)
Inactive	23 (52)
No data	2 (5)
IBD treatment at time of CRC diagnosis	
None	10 (22)
1 drug	13 (30)
2+ drugs	13 (30)
No data	8 (18)
Primary sclerosing cholangitis	6 (14)
IBD - surgery interval (years)	21.5 ± 12.6

Values presented as mean ± standard deviation or number  (column %).

**Figure 1.**
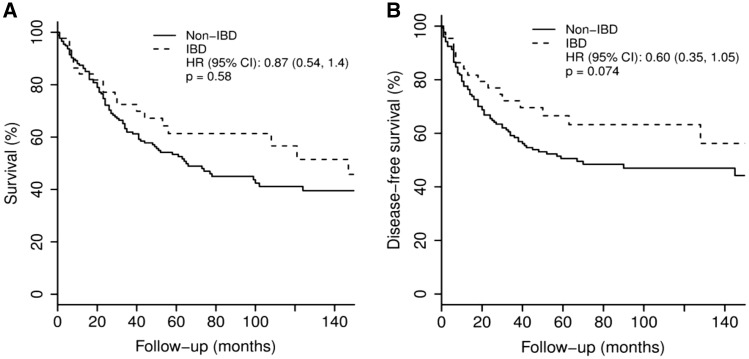
Overall survival (**A**) and disease-free survival (**B**) following postoperative chemotherapy in colorectal cancer patients with and without inflammatory bowel disease.

Crude mortality was observed in 52% of IBD-associated CRC patients (mean follow-up: 74.1 ± 58.9 months) and 49% of non-IBD CRC patients (mean follow-up: 64.8 ± 71.0 months) (*P* = 0.58). There was no significant difference in disease-free survival (HR = 0.60; 95% confidence interval [CI]: 0.35–1.05; *P* = 0.074) or overall survival (HR = 0.87; 95% CI: 0.54–1.4; *P* = 0.58) between IBD and non-IBD patients (**Figure 1**).

After adjusting for the propensity score, there was a trend toward significance (*P* = 0.060) suggesting that IBD patients might have a better disease-free survival rate than non-IBD patients. **Table 3** compares disease-free survival and overall survival based on the IBD status and degree of tumor differentiation.

**Table 3.** Survival outcomes of CRC patients following postoperative chemotherapy: multivariable Cox regression analysis

	Overall survival		Disease-free survival	
	HR (95% CI)	*P* value	HR (95% CI)	*P* value
IBD *vs* non-IBD	0.87 (0.53–1.4)	0.58	0.57 (0.32–1.02)	0.060
Grade of differentiation (1 grade increase)	1.3 (0.85–1.9)	0.23	1.6 (1.02–2.5)	0.041

Multivariable Cox regression was used to analyze overall survival, and competing risks analysis was used to analyze disease-free survival, censoring subjects who died from other causes or remained alive at end of follow-up.

## DISCUSSION

IBD-associated CRC does not necessarily follow the typical adenoma-to-cancer sequence of events as most cases of sporadic CRC [11]. The presence, degree, duration and anatomical extent of colonic inflammation have been associated with the risk of CRC in patients with IBD [12]. Chronic mucosal inflammation is thought to result in gradual progression to dysplasia and ultimately invasive CRC. A number of genomic disruptions have been identified within cells from colonic mucosa of patients having chronic colitis including aneuploidy, aberrant DNA methylation and p53 mutations [10,13,14].

Prognosis of patients with IBD-associated CRC has been variably reported; some reports showed a prognosis comparable to sporadic CRC [15–17], while other reports suggested an increased risk of death and decreased overall survival [18–23]. For example, in one report, stage III CRC patients with IBD in particular showed significantly decreased survival (23.0 *vs* 133.9 months for patients with sporadic CRC), although most patients in that study received chemotherapy with or without radiation [23]. However, a direct comparison of tumor recurrence and survival rates following postoperative chemotherapy in CRC patients with and without IBD has not been previously reported.

In a prior study from our group, we demonstrated morphologic similarity between IBD-associated CRC and microsatellite instability high (MSI-H) CRC [24]. Sporadic MSI-H CRC is less responsive to fluorouracil treatment compared with sporadic microsatellite stable (MSS) CRC [24]. In addition, patients with IBD have been reported to have high risk of intestinal toxicity (e.g. diarrhea) from fluorouracil-based chemotherapy [25]. Furthermore, the presence of PSC in patients with IBD-associated CRC may result in increased risk of liver toxicity specifically derived from fluorouracil-based chemotherapy. Thus, our initial hypothesis was that patients with IBD-associated CRC may have less response to fluorouracil-based chemotherapy and thus worse prognosis than patients with sporadic CRC.

In this matched case-control study, there were no differences in tumor recurrence rate, disease-free survival and overall survival following postoperative chemotherapy in CRC patients with and without IBD. These findings suggest that fluorouracil-based chemotherapy is effective in IBD-associated CRC despite its morphologic resemblance to MSI-H CRC. This seemingly contradictory result may be explained by MSI-H histologic morphology of IBD-associated CRC being independent of MSI status [24].

This is the largest study to date examining tumor recurrence, disease-free survival and overall survival in patients with IBD-associated CRC in comparison with a matched large control group of sporadic CRCs. A major strength of this study is that all IBD-associated CRC cases were confirmed by histologic review rather than clinical diagnosis alone. Limitations of the study include its retrospective design and the exclusion of 21 patients due to incomplete clinical data. As with all retrospective studies, data can be limited by the quality and availability of the information found within the database and medical records. In particular, although a fluorouracil-based regimen is recorded in our prospectively maintained CRC database, more detailed information regarding dosage, use of radiation and toxicity were lacking in several patients. Specific details of oxaliplatin use were not available since we included CRC patients diagnosed and treated before and after 2004, when oxaliplatin was added to fluorouracil regimens for stage II/III CRC. However, prior clinical trials showed that oxaliplatin affects disease-free survival only and not overall survival of those patients [26], thereby minimizing the impact of this limitation on our results.

In summary, our study showed no difference in tumor recurrence, disease-free survival and overall survival in patients with IBD-associated CRC compared with non-IBD CRC patients following surgical resection and postoperative chemotherapy. These results suggest that, as in sporadic CRC patients, those with IBD-associated CRC should be considered for postoperative chemotherapy after surgical resection if otherwise eligible for such treatments. Large prospective studies are needed to confirm these findings.

## Author contributions

Mohannad Dugum and Xiuli Liu: study concept and design, acquisition of data, analysis, data interpretation, drafting of the manuscript and critical revision of the manuscript for important intellectual content. Rocio Lopez: statistical analysis, interpretation of data and drafting of the manuscript. Jingmei Lin, Bassam Estfan, Elena Manilich, Luca Stocchi and Bo Shen: acquisition of data and critical review of manuscript for important intellectual content. All authors approved the final version of the manuscript.

*Conflicts of interest statement:* none declared.
